# Endogenous Morphine in SH-SY5Y Cells and the Mouse Cerebellum

**DOI:** 10.1371/journal.pone.0001641

**Published:** 2008-02-20

**Authors:** Arnaud Muller, Elise Glattard, Omar Taleb, Véronique Kemmel, Alexis Laux, Monique Miehe, François Delalande, Guy Roussel, Alain Van Dorsselaer, Marie-Hélène Metz-Boutigue, Dominique Aunis, Yannick Goumon

**Affiliations:** 1 Inserm, U575, Physiopathologie du Système Nerveux, Strasbourg, France; 2 Faculty of Medicine, Institut de Chimie Biologique, Strasbourg, France; 3 Centre National de la Recherche Scientifique (CNRS), Laboratoire de Spectrométrie de Masse Bio-Organique, The European School of Chemistry, Polymers and Materials (ECPM), Université Louis Pasteur, LC4-UMR7178, Strasbourg, France; Fred Hutchinson Cancer Research Center, United States of America

## Abstract

**Background:**

Morphine, the principal active agent in opium, is not restricted to plants, but is also present in different animal tissues and cell types, including the mammalian brain. In fact, its biosynthetic pathway has been elucidated in a human neural cell line. These data suggest a role for morphine in brain physiology (*e.g*., neurotransmission), but this hypothesis remains a matter of debate. Recently, using the adrenal neuroendocrine chromaffin cell model, we have shown the presence of morphine-6-glucuronide (M6G) in secretory granules and their secretion products, leading us to propose that these endogenous alkaloids might represent new neuroendocrine factors. Here, we investigate the potential function of endogenous alkaloids in the central nervous system.

**Methodology and Principal Findings:**

Microscopy, molecular biology, electrophysiology, and proteomic tools were applied to human neuroblastoma SH-SY5Y cells (*i*) to characterize morphine and M6G, and (*ii*) to demonstrate the presence of the UDP-glucuronyltransferase 2B7 enzyme, which is responsible for the formation of M6G from morphine. We show that morphine is secreted in response to nicotine stimulation *via* a Ca^2+^-dependent mechanism involving specific storage and release mechanisms. We also show that morphine and M6G at concentrations as low as 10^−10^ M are able to evoke specific naloxone-reversible membrane currents, indicating possible autocrine/paracrine regulation in SH-SY5Y cells. Microscopy and proteomic approaches were employed to detect and quantify endogenous morphine in the mouse brain. Morphine is present in the hippocampus, cortex, olfactory bulb, and cerebellum at concentration ranging from 1.45 to 7.5 pmol/g. In the cerebellum, morphine immunoreactivity is localized to GABA basket cells and their termini, which form close contacts on Purkinje cell bodies.

**Conclusions/Significance:**

The presence of morphine in the brain and its localization in particular areas lead us to conclude that it has a specific function in neuromodulation and/or neurotransmission. Furthermore, its presence in cerebellar basket cell termini suggests that morphine has signaling functions in Purkinje cells that remain to be discovered.

## Introduction

Morphine is one of the 40 alkaloids present in opium from *Papaver somniferum*, and is one of the strongest known analgesic compounds [1]. Endogenous morphine has been characterized in numerous mammalian cells and tissues [2], [3], [4], and its structure is identical to that of morphine from poppy (for review see [1], [5]). In mammals, the biosynthesis of endogenous morphine is associated with that of dopamine and catecholamines [5], [6], [7], [8], [9], [10]. Morphine biosynthesis was recently shown in the SH-SY5Y human neuronal catecholamine-producing cell line [8], [9], a well-known model for studying neuronal secretion [11], [12]. Recently, our group has reported the presence of morphine-6-glucuronide (M6G), previously considered a product of morphine catabolism, in the secretory granules and secreted material of bovine adrenal chromaffin cells. In these cells, M6G represents the final product of endogenous alkaloid biosynthesis and is formed through the action of an UDP-glucuronosyltransferase 2B-like enzyme (UGT2B). In chromaffin granules, M6G is bound to the intragranular phosphatidylethanolamine-binding protein (PEBP or Raf kinase inhibitor protein) [13], which also binds morphine [14]. Secretion of M6G, catecholamines, and PEBP [15] into the blood is likely to occur during stress situations, and could be involved in different stress-modulating or pain-modulating mechanisms *via* binding to µ opioid receptors (MORs), which are present on numerous cell types [16], [17], [18]. Together, all these observations suggest that endogenous alkaloids may represent new neuroendocrine factors [15].

Endogenous morphine has been detected in the brains of cows, rats, monkeys, and dogs (for review, see [1]). However, the function of endogenous morphine in the brain remains unknown, and knowledge of its distribution in cerebral areas and neuronal cells is lacking. Enzymes such as those in the UGT1A family, implicated in converting morphine to inactive M3G, are expressed in rat primary neurons and astrocytes [19]. UGT2B7, which produces M6G and M3G, is also expressed in the human brain [20]. Interestingly, human and rat brain extracts incubated with radiolabeled morphine produce labeled M3G and M6G [21], [22]. Nevertheless, it remains a matter of debate whether morphine or its derivatives function as neuromediators and/or neurotransmitters.

The present study addresses the function of morphine in the brain. Microscopy, biochemistry, cell biology, molecular biology, and electrophysiology have been used to characterize morphine and its derivatives in detail in the human SH-SY5Y neuroblastoma cell model. At the ultrastructural level, morphine immunoreactivity colocalizes with that of chromogranin A (CGA), a well-known marker of Large Dense Core (LDC) vesicles. We have found that the UGT2B7 enzyme, which converts morphine to M6G, is expressed in these cells. Upon nicotine stimulation, morphine is released from SH-SY5Y cells *via* a Ca^2+^-dependent mechanism. Experiments using the patch-clamp technique reveal that naloxone-sensitive electrophysiological responses are induced at concentration of morphine and M6G as low as 10^−10^ M. To extend these experiments in cultured cells, we quantified morphine levels in different areas of the mouse brain. In the cerebellum, morphine immunoreactivity is concentrated in the basket cells and their termini, which innervate Purkinje cell bodies. This study documents the presence of morphine in the brain and shows a specific localization that implies a signaling role that is yet to be precisely established.

## Results

Human neuroblastoma SH-SY5Y cells synthesize endogenous morphine [9] and express MORs [17], [23], [24]. Therefore, we used this cell line to determine the subcellular localization, biosynthesis, and secretion of morphine.

### Subcellular localization of morphine in SH-SY5Y cells

**Figure 1:**
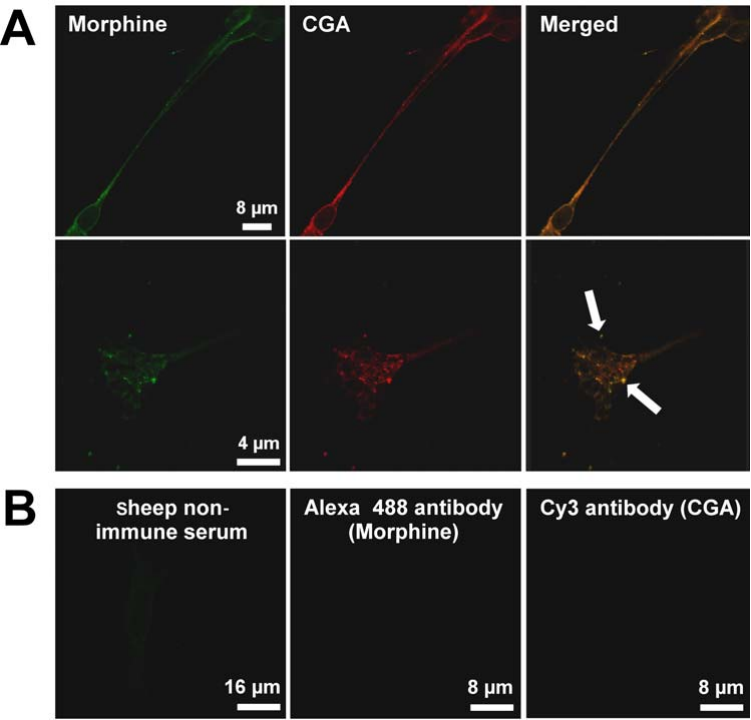
Evidence of the presence of morphine-like immunoreactivity in secretory granules. *A*. *Upper panel*, double immunofluorescence confocal micrographs. Labeling was performed with a sheep anti-morphine antibody (visualized in green with an Alexa Fluor 488-conjugated IgG) and with an antibody against CGA (a specific intragranular marker) visualized in red with a mouse Cy3-conjugated IgG. Colocalized immunolabelling (merged window) appears as yellow staining. *Lower panel*, SH-SY5Y termini shown at higher magnification. Arrows indicate colocalization points. An identical pattern of labelling was obtained for a different mouse monoclonal anti-morphine antibody (data not shown). *B.* To assess the specificity of morphine immunolabelling, control experiments were performed using either sheep non-immune serum and an Alexa Fluor 488-conjugated IgG, or Alexa Fluor 488-conjugated IgG and Cy3-conjugated IgG without a primary antibody.

Using laser confocal microcopy, the labeling obtained with an anti-morphine sheep polyclonal antibody was compared to that of CGA [15], a specific marker of LDC vesicles [25], [26], [27], [28]. Anti-morphine immunolabeling showed a punctate pattern in the cytoplasm, similar to that obtained with the anti-CGA antibody (Fig. 1A). Superimposition of the two labelings reveals an intravesicular colocalization (Fig. 1A, yellow label). At higher magnification, anti-morphine immunolabeling was also visible in neurite termini, and remained clearly colocalized with CGA (Fig. 1A, lower panel, arrows). Control experiments established the specificity of the labeling in vesicles using either the secondary antibody alone, non-immune sheep serum (Fig. 1B), or the anti-morphine antibody blocked with morphine prior to its use in immunocytochemistry experiments [15].

### Evidence for the presence of UGT2B7 in SH-SY5Y cells

**Figure 2:**
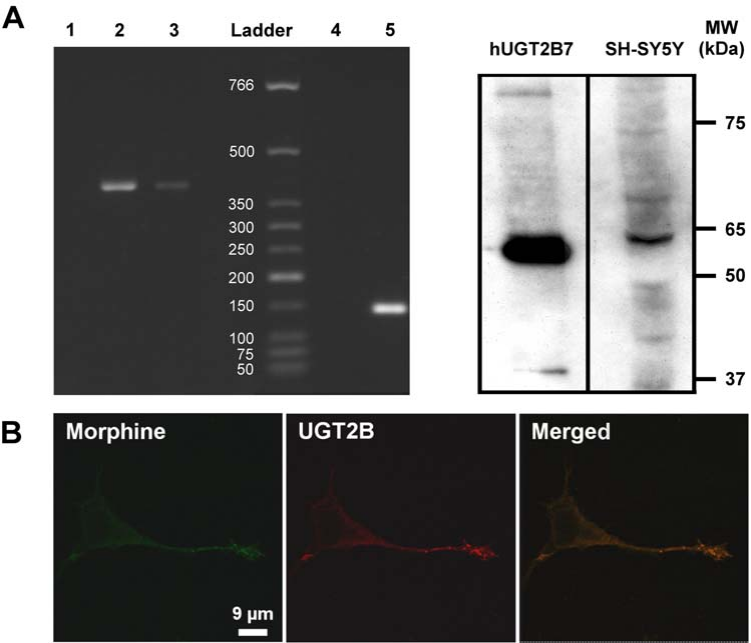
Evidence for UGT2B7 in SH-SY5Y cells. *A*. Amplification of UGT2B7 RNA and Western blot analysis. *Left panel*, total SH-SY5Y RNA was submitted to RT-PCR using specific human UGT2B7 primers. Lane 1, control using water to test for contamination. Lane 2, total RNA from human hepatocyte extract was used as a positive control, showing a single band of 462 bp corresponding to the expected UGT2B7 PCR product. Lane 3, SH-SY5Y total RNA showing a single band of 462 bp. Lane 4, negative control of GAPDH amplification (water). Lane 5, SH-SY5Y total RNA (GAPDH, 142 bp). The size of standards are indicated in bp. *Right panel*, Western blot analysis of SH-SY5Y cell extracts was done using 1 µg of human recombinant UGT2B7 (positive control) and 50 µg of SH-SY5Y cell extract. Western blots using anti-UGT2B antibody show a band at 65 kDa [15]. *B*. Localization of UGT2B immunoreactivity in SH-SY5Y cells. Double immunofluorescence confocal micrographs were obtained using an anti-UGT2B antibody (detected in red with an Alexa Fluor 568-conjugated IgG) and with an antibody against morphine visualized in green with an Alexa Fluor 488-conjugated IgG. Colocalized immunolabelling appears as yellow staining (merged window).

The next step was to examine whether SH-SY5Y cells have the capacity to synthesize M6G. First, a PCR approach was used to determine whether UGT2B7, the only enzyme known to convert morphine into M6G, is expressed in SH-SY5Y cells. As shown in Figure 2A, amplification of UGT2B7 RNA performed on SH-SY5Y total RNA extracts indicates the presence of a single band at 462 bp, which corresponds to the band present in total RNA from human primary hepatocytes.

These experiments were then complemented by Western blot analysis of SH-SY5Y cell extracts in order to detect UGT2B protein expression (Fig. 2A). Using recombinant human UGT2B7 as a positive control (Fig. 2A), UGT2B-like immunoreactivity was observed as a band at 65 kDa, showing, for the first time, that SH-SY5Y cells may be capable of synthesizing M6G.

The subcellular localization of the UGT2B7 enzyme was examined by laser confocal immunocytochemistry and compared to that of morphine and CGA. The punctuate labelling of UGT2B overlapped with that of morphine (Fig. 2B, yellow label), revealing the presence of UGT2B in LDC vesicles. These results indicate that the M6G-producing enzyme UGT2B7 is expressed in SH-SY5Y cells, and that it colocalizes with morphine.

### Characterization of morphine and M6G in SH-SY5Y cells

**Figure 3:**
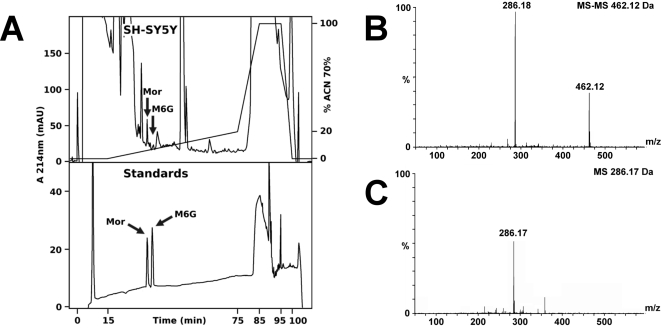
Characterization of morphine and M6G in SH-SY5Y extracts. *A.* Purification of morphine and M6G. Upper panel, RP-HPLC chromatogram showing the purification of endogenous alkaloids from SH-SY5Y cells (375×10^6^ cells). Lower panel, RP-HPLC purification of morphine (Mor) and M6G standards (500 pmol). *B.* Characterization of M6G. Q-TOF MS-MS analysis of the HPLC fraction showing M6G in the intragranular material (marked with an arrow in Fig. 3A, upper panel). The fragment at 462.12 Da corresponds to M6G, whereas the 286.18 Da fragment corresponds to morphine. *C.* Q-TOF MS analysis of the HPLC fraction showing morphine (286.17 Da) in the intragranular material (marked with an arrow in Fig. 3A, upper panel).

After showing that the UGT2B-like enzyme is present in SH-SY5Y cells, we sought to confirm the presence of morphine and M6G in the cells using mass spectrometry (MS). SH-SY5Y extracts were purified by HPLC (high performance liquid chromatography) using a specific gradient designed to separate morphine and M6G. Comparison with an elution profile of alkaloid standards (Fig. 3A) allowed for the identification of fractions containing endogenous morphine and M6G. Q-TOF MS-MS analysis of material present in the corresponding fractions unambiguously identified both morphine (m/z = 286.13Da; Fig. 3C) and M6G (m/z = 462.15Da; Fig. 3B). Together, these data confirm the presence not only of endogenous morphine, but also of M6G in SH-SY5Y cells.

### Morphine secretion from SH-SY5Y cells

**Figure 4:**
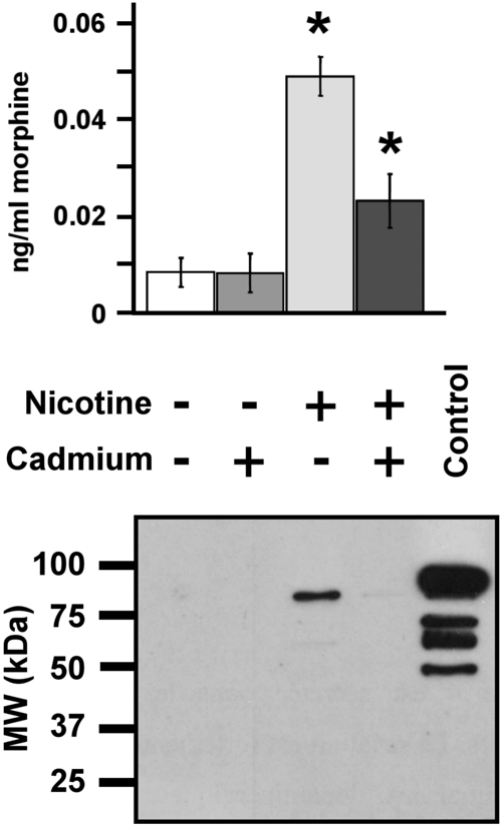
Characterization of morphine secretion from SH-SY5Y cells. *Upper panel*. The amount of morphine secreted into the culture medium was determined after stimulating 4×10^5^ cells with 100 µM of nicotine with or without cadmium (200 µM) for 24 h. The basal secretion level was obtained from cells incubated without nicotine at the same time (n = 6). Amounts of secreted morphine in the nicotine and nicotine+cadmium groups were statistically different and were both different from the two control groups (no nicotine and no nicotine+cadmium; Mann-Whitney test, * p<0.01). *Lower panel*. The efficiency of secretion was assessed by monitoring the secretion of chromogranin B (CGB), an intra-LDC vesicle protein [28], by Western blot analysis. A positive control, intragranular protein matrix from bovine chromaffin cells (10 µg), was loaded in order to evaluate the molecular weight of the entire CGB (80 kDa).

In order to quantify morphine secretion from SH-SY5Y cells and to determine whether it occurs *via* a Ca^2+^-dependent mechanism, cells were stimulated with 100 µM nicotine to trigger membrane depolarization and LDC vesicle exocytosis [28]. The amount of morphine released was measured using a specific and sensitive ELISA technique. A low basal level of morphine secretion was observed in the absence of nicotine (Fig. 4). Stimulation of SH-SY5Y cells with 100 µM nicotine induced a five-fold increase in secreted morphine over background (Fig. 4). In the presence of the Ca^2+^-channel blocker cadmium, basal secretion remained unchanged, while nicotine-induced secretion of morphine decreased dramatically. These results unambiguously indicate that morphine can be released *via* a Ca^2+^-dependent mechanism upon stimulation of SH-SY5Y cells.

In order to identify the origin of stored morphine, the release of chromogranin B (CGB), an intragranular marker of SH-SY5Y LDC vesicles [28], was investigated by Western blot analysis (Fig. 4). The presence of an 80 kDa CGB-immunoreactive band was detectable among proteins in the extracellular medium when cells were treated with nicotine. Similar to morphine, nicotine-evoked CGB secretion also dramatically decreased when cadmium was present in the medium (Fig. 4). In conclusion, SH-SY5Y cells secrete morphine *via* a Ca^2+^-dependent mechanism in response to nicotine stimulation.

### Evidence for physiological effects of morphine and M6G on SH-SY5Y cells

**Figure 5:**
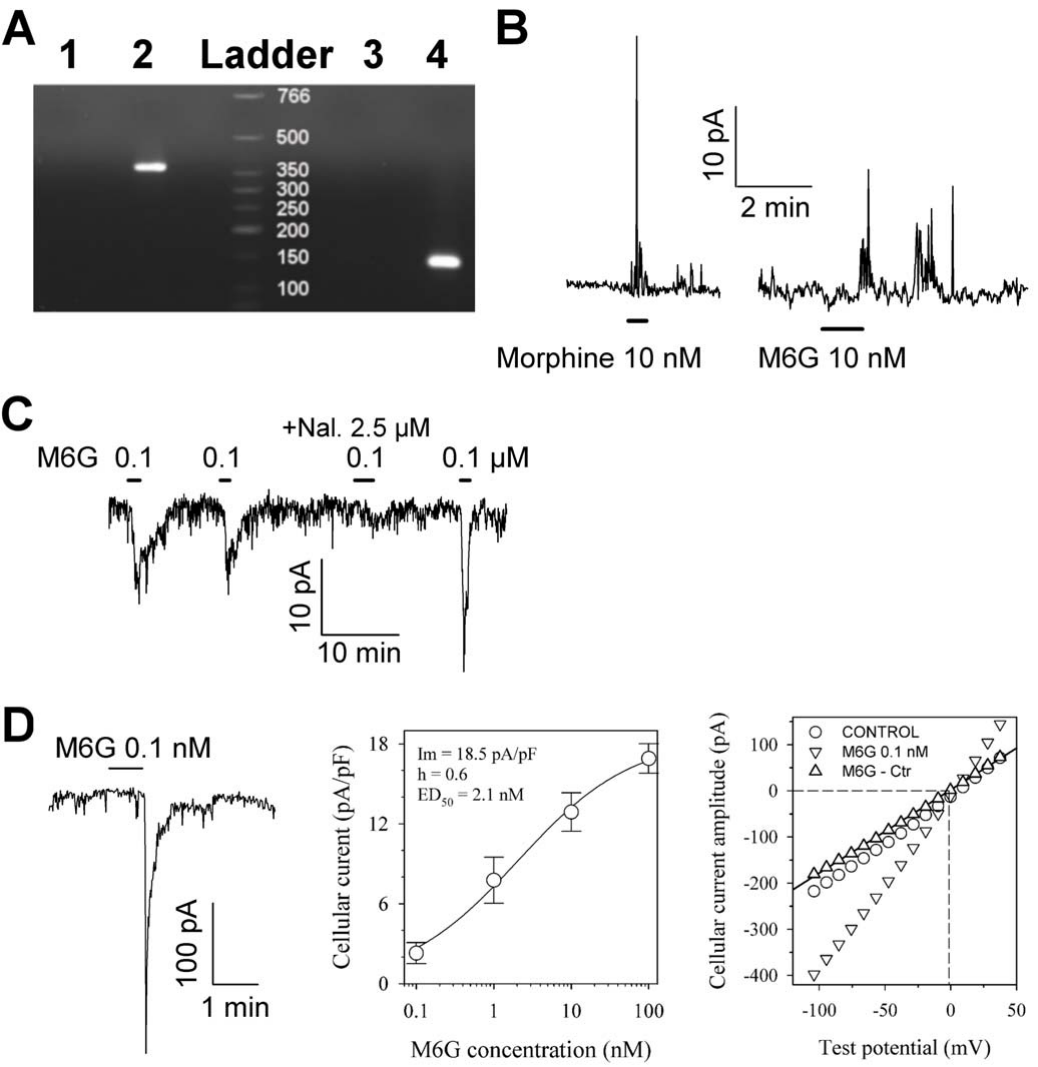
Amplification of MOR1 RNA and characterization of the electrophysiological effects of a low concentration of morphine and M6G. *A*. Total SH-SY5Y RNA was submitted to RT-PCR using specific human MOR1 primers. Lane 1, control using water as a PCR template to test for contamination. Lane 2, total RNA from SH-SY5Y, showing a single band of 376 bp. Lane 3, negative control of GAPDH amplification (water). Lane 4, SH-SY5Y total RNA (GAPDH, 142 bp). Size standards are indicated in bp. *B* and *C.* Typical responses of SH-SY5Y cells to low concentrations of morphine and M6G antagonized by naloxone, as measured by the patch clamp technique in cell-attached mode at a pipette potential of 60 mV and 80 mV (*B* panel), as well as at −135 mV (*C* panel). M6G was repeatedly applied in the absence or presence of naloxone (Nal.). *D*. Whole-cell patch clamp recording of the M6G response: the left-hand panel shows a typical trace recorded at a holding potential of −135 mV, the middle panel shows a dose-response curve obtained at a holding potential of −80 mV, and the right-hand panel shows an I-V plot of the peak amplitude response. The dose-response data were fitted with Hill's equation (continuous line) with optimal parameters as indicated in the Figure. The I-V plot of the steady state current for the control (circles) and at the peak of the response to 0.1 nM M6G (inverted triangles) was obtained using a ramp potential protocol from 80 to −140 mV lasting 800 ms. The specific current elicited in the presence of M6G (upright triangles) was linear, and the reversal potential calculated by linear regression gave a value of −0.5 mV (dashed line). Traces were filtered at 2 kHz and digitized at 5 kH. Bars indicate the period of drug application at the indicated concentrations.

To address whether morphine and M6G induce a physiological response in SH-SY5Y cells, we first looked at the expression of the µ opioid receptor family (MOR1; [17], [18], [29], [30]). Using SH-SY5Y total RNA library, we detected MOR1 transcripts using PCR (Fig. 5A, 376 bp band), suggesting that SH-SY5Y cells are responsive to morphine or M6G.

In light of these results, we examined the response of SH-SY5Y cells to alkaloids using the patch clamp electrophysiological approach. As shown in Figure 5, SH-SY5Y cells were responsive to M6G and morphine in either the cell-attached or the whole-cell configuration. Approximately 70% of the 26 randomly chosen cells were responsive.

The cellular response to M6G application was fully reproducible in about 80% of positive cells (Fig. 5C). However, 20% of the positive cells responded to a first M6G application, but not to subsequent applications even after one hour of washout, suggesting a strong desensitization in these cells. M6G was active in SH-SY5Y cells at doses as low as 0.1 nM M6G (Fig. 5D). The cellular response in whole-cell recording corresponded to a membrane potential of −80 mV and an inward current, the peak amplitude of which was dose-dependent. Dose-response curves were obtained for 13 different cells tested with M6G at concentrations ranging from 0.1 to 100 nM. The peak amplitude cellular response was normalized to the cell surface area in order to reduce variability due to cell size. Application of Hill's model to the dose-response data yielded an ED_50_ value of 2.1 nM and a slope of 0.6 (Fig. 5D, middle panel). The I-V plot obtained at the peak cellular response to 0.1 nM M6G was linear. Linear regression analysis of the specific current data in the presence of M6G yielded a calculated reversal potential of about −0.5 mV (Fig. 5D, right-hand panel, upright triangles). This value was close to the equilibrium potential (E_cat_ = 0 mV) for monovalent cations, indicating that M6G activates a channel that allows for the passage of Na and K ions. Figure 5C illustrates the sensitivity of the M6G response to the opioid receptor antagonist naloxone. When used at a concentration of 2.5 µM, naloxone inhibited 90% of the cellular response to 0.1 µM M6G. Morphine produced similar effects when applied at low concentration (Fig. 5B).

These data indicate that morphine and M6G can evoke specific membrane currents at low concentrations *via* opioid receptors, indicating a possible regulatory role for secreted morphine/M6G in SH-SY5Y cell culture.

### Immunomapping of endogenous morphine in the mouse brain

The presence in SH-SY5Y cells of endogenous alkaloids in LDC vesicle-like structures, their release by a Ca^2+^-dependent exocytotic mechanism, and their effects on these cells at concentration as low as 0.1 nM led us to investigate whether endogenous morphine may represent a neuromodulator in the mouse brain, where M6G is absent [31].

**Figure 6:**
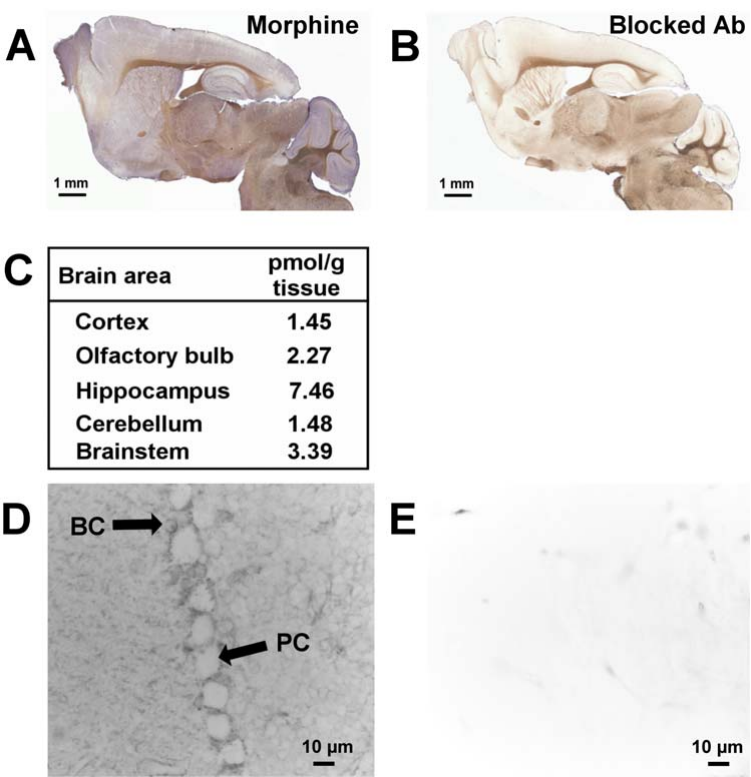
Mapping of endogenous morphine in the mouse brain. *A*. Immunodetection of morphine present in the mouse brain. Sagittal slices were incubated with a mouse monoclonal anti-morphine antibody and visualized with an HRP-conjugated donkey anti-mouse IgG, in order to detect endogenous morphine in specific brain areas. *B.* Control experiment using morphine immunoadsorbed mouse monoclonal antibody (same incubation time as in *A*). *C.* Quantification of the morphine present in different mouse brain areas using morphine-specific ELISA. The table shows the quantities of endogenous morphine (pmoles) present per gram of wet tissue. The values correspond to an average of the morphine amount determined for 5 brains (n = 5). *D*. Localization of morphine label in the cerebellum using mouse monoclonal anti-morphine antibody and an HRP-conjugated secondary antibody. Morphine labelling was observed around Purkinje cells and in basket cells. PC, Purkinje cell; BC, basket cell. *E.* Control for immunolabelling using morphine-immunoadsorbed mouse monoclonal antibody.

**Figure 7:**
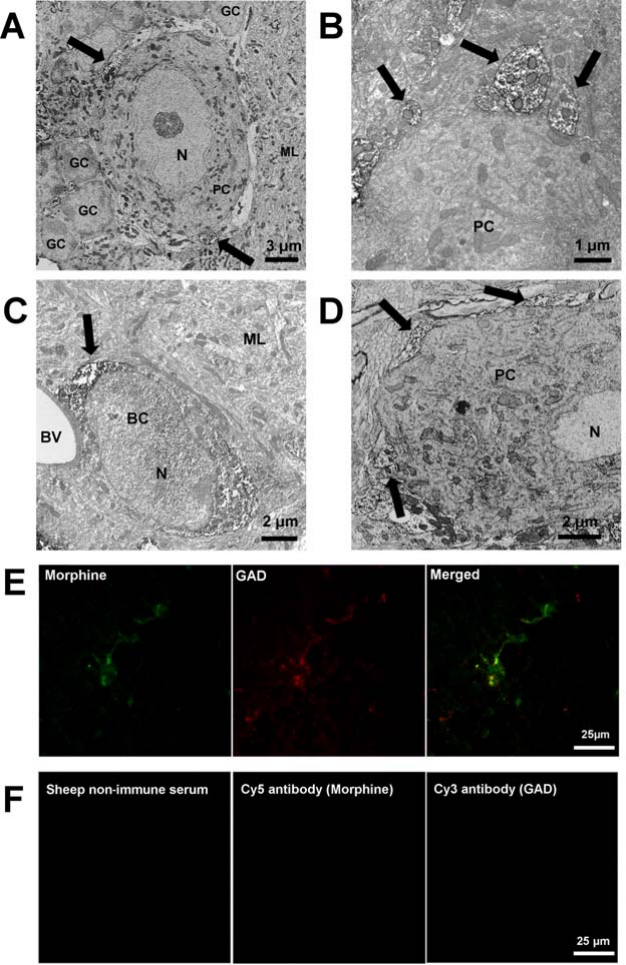
Characterization of morphine immunoreactivity in the mouse cerebellum. *A*. Characterization of morphine immunolabelling around Purkinje cells. Electron microscopy using sheep anti-morphine antibody showed morphine-like immunoreactivity in nerve termini innervating Purkinje cell bodies (PC). Arrows indicate strong immunolabelling of morphine-containing termini around the Purkinje cell body. GC, granular cell. ML, molecular layer, N, nucleus. *B.* Higher magnification showing the presence of morphine immunoreactivity in nerve termini innervating Purkinje cell bodies. *C.* Electron microscopy showing morphine immunoreactivity in basket cells (BC). BV, blood vessel. ML, molecular layer. Control experiments using anti-sheep HRP-conjugated IgG showed the specificity of the immunolabelling. *D.* Electron microscopy showing PEBP-immunoreactivity in nerve termini covering Purkinje cell bodies (PC). Arrows indicate strong immunolabelling of PEBP-containing basket cell termini. *E.* Evidence of colocalization of morphine and glutamic acid decarboxylase (GAD). Double immunofluorescence confocal micrographs were obtained using a sheep anti-morphine antibody (detected with a CY5-conjugated IgG, green pseudocolor label) and an antibody against GAD visualized in red with a Cy3-conjugated IgG. Colocalized immunolabelling (arrows and merged window) appears as yellow staining. *F.* Control experiments were performed using sheep non-immune serum, or only secondary antibodies detected with Cy5- and Cy3-conjugated IgG (primary antibody omitted), to demonstrate the specificity of the immunolabelling.

The localization of endogenous morphine was examined in the mouse CNS by immunocytochemistry using an anti-morphine mouse monoclonal antibody (6D6 antibody). Light microscopy revealed the presence of morphine immunolabelling in well-defined brain structures such as the cerebellum, the olfactory bulb, the hippocampus, and the cortex (Fig. 6A). Fainter, dispersed labelling was seen in other structures. Quantification of morphine levels in these areas was performed using a morphine-specific ELISA kit. The higher amount of morphine (7.46 pmol/g of fresh tissue) was present in the hippocampus (Fig. 6C; n = 5). We chose to focus on the cerebellum area in greater detail because of the apparently clear-cut Purkinje cell labelling (Fig. 6A) and the concomitant presence of MORs in these cells [32], [33]. Conventional microscopy, however, revealed that the labelling was not within Purkinje cells themselves, but rather in synaptic endings in contact with Purkinje cells (Fig. 6D, PC). Immunolabelling was observed in basket cells close to Purkinje cells (Fig. 6D, BC). In addition, discrete morphine immunolabelling could be observed in the white matter afferent fibers and in some isolated cells (data not shown). Synapses innervating the Purkinje cell bodies originate from basket cells known as inhibitory GABA interneurons, which are located in the molecular layer close to the Purkinje cells. Electron and laser confocal microscopy were used to determine whether labelling was present in basket cells:

(*i*) Electron microscopy experiments showed strong immunolabelling of nerve termini over the entire Purkinje cell body (Fig. 7A & 7B). These endings originate from basket cells which were immunoreactive to the morphine antibody (Fig. 7C).

In parallel, the immunoreactivity of the morphine-binding protein PEBP [14] was found in basket cell nerve termini around Purkinje cell bodies (Fig. 7D, arrow), suggesting the presence of a morphine-PEBP complex in these structures.

(*ii*) Confocal microscopy experiments using both anti-morphine and anti-glutamic acid decarboxylase antibodies revealed the colocalization of morphine and glutamic acid decarboxylase around the Purkinje cell bodies (Fig. 7E, yellow label on the merge window). Appropriate controls confirmed the specificity of this immunolabelling (Fig. 7F).

## Discussion

The significance of the detection of endogenous morphine in the brains of rats, mice, monkeys, and cows remains a matter of debate as long as its functional role is unknown [1], [34]. Previous studies have shown that radiolabelled morphine is taken up and secreted by brain slices and immune cells [22], [35], [36], [37]. Further study has linked levels of endogenous morphine in the brain to physiological states (*e.g.*, inflammation and fasting) [7], [38], [39], [40]. However, a thorough investigation to determine whether endogenous morphine acts as a true neuromodulator has not yet been published. The aim of the present study was to begin to examine this question and thereby take the first steps towards establishing a functional role for endogenous morphine.

To be considered a neurotransmitter, endogenous morphine should (***i***) be present in nerve terminals and stored within secretory vesicles, (***ii***) possess a specific pre/postsynaptic receptor able to induce specific effects (*i.e*., MOR), (***iii***) be released upon depolarizing stimulation *via* a Ca^2+^-dependent mechanism, and (***iv***) be degraded or recycled by an extra- or intracellular mechanism. We examined these criteria using the neuronal SH-SY5Y cell line and mouse brain.

### (*i*) Presence of morphine in secretory granules and nerve termini

The human neuroblastoma clone SH-SY5Y, derived from a human sympathetic ganglion [41], possesses the properties of sympathetic neurons [42] and is a well-established model for studying the secretion of neurotransmitters [11], [12], [28]. These cells have been shown to express several types of receptors, including MOR and δ opioid receptor subtypes (DOR) [16], [17], [18]. SH-SY5Y cells are characterized by the presence of numerous LDC vesicles (100–200 nm in diameter) which are usually found in sympathetic neurons and that closely resemble the adrenal chromaffin secretory granules. These LDC vesicles store neurotransmitters such as noradrenaline, enkephalins, and chromogranins. Morphine synthesis has been observed in SH-SY5Y cells [9]. The present results reveal that morphine immunoreactivity (which identifies morphine and M6G) is present within secretory structures, as shown by its colocalization with CGA. The colocalization in LDC vesicle-like structures occurs in cell bodies but also along cell processes and within neurite endings.

Biochemical analyses using the sensitive technique of mass spectrometry confirmed the presence not only of morphine in SH-SY5Y cell extracts, but also of M6G, a product of morphine catabolism that has never been found in this cell type [43], [44]. We recently reported the presence of M6G in the matrix of bovine adrenal chromaffin secretory granules, as well as in the material secreted from chromaffin cells [15]. We were able to show that in chromaffin cells, M6G is a product of anabolic synthesis by UDP-glucuronosyltransferase 2B-like enzyme (UGT2B). However, the UGT2B7 detected here in SH-SY5Y cells is probably responsible for M6G biosynthesis. While in chromaffin cells morphine is almost completely metabolized into M6G, we found that morphine and M6G coexist in SH-SY5Y cells, suggesting that the two molecules are cosecreted *in vivo*.

Using an immunohistochemical approach, morphine was detectable in the mouse CNS, particularly in specific neurons and nerve terminals. A strong localized immunoreactivity was observed in the cerebellum, olfactory bulb, hippocampus, and cortex, whereas other structures showed more dispersed labelling. Faint immunolabelling was also visible in the white matter, and this may correspond to morphine-positive afferent fibers. Using optical methods, we observed intense immunostaining in the cerebellum surrounding Purkinje cell bodies and in basket cells. Ultrastuctural techniques showed morphine immunoreactivity to be concentrated in the cell bodies of basket cells and their endings that make close contacts with Purkinje cell bodies. These anatomical findings implicate this endogenous alkaloid in cerebellum activity.

### (*ii*) Presence of a specific pre/postsynaptic receptor

We investigated whether SH-SY5Y cells respond to the application of morphine and M6G. First, we confirmed that they express MOR mRNA [23], [24]. MORs belong to a large family of receptor proteins derived from the *OPRM* (opioid receptor µ) gene. MORs are membrane proteins with seven transmembrane domains, and they are coupled to G_o/i_ proteins (for review, see [45]). Stimulation of these receptors leads to various effects, including analgesia and regulation of the gastrointestinal tract. The distribution of MORs in the mouse brain has previously been investigated using immunohistochemistry and molecular biology [46]. However, this is a complex problem given the multiplicity of splice variants, with 32 mRNA splice forms and at least 11 receptor isoforms for mouse MOR1 [1], [45], [47]. MOR1 subtypes have been described in mouse, rat, and cat Purkinje cells [32], [33], [48], [49].

The presence of morphine in cerebellum basket cells that form synaptic contacts with Purkinje cell bodies led us to speculate that endogenous morphine may be involved in modulating Purkinje cell output. It is well known that GABA liberated from basket cell endings modulates Purkinje cell activity. Moreover, DAMGO and baclofen, two MOR agonists, have been reported to regulate non-activating outward currents in mouse Purkinje cells when used at µM concentrations [50]. Interestingly, DAMGO inhibits GABA neurotransmission [51]. In the case of morphine, microiontophoretic application to cat Purkinje cells induces either excitatory or inhibitory responses (*i.e*., an increase or decrease of the firing rates, respectively) [48], [49]. Naloxone reverses excitatory morphine-evoked actions, whereas inhibitory effects can be reversed only by the GABA-receptor antagonist bicuculine. This suggests that the excitation of cat Purkinje cells by morphine is related to MORs, whereas inhibitory events involve GABA receptors. Whether morphine and GABA are stored in the same vesicles and released together from basket cells remains an open question. The presence of both molecules in the nerve endings of the same cell may imply complex regulatory processes yet to be elucidated.

### (*iii*) Secreted morphine

At least 11 mouse MOR1 isoforms exist [47] and have various affinities for morphine and its derivatives (K_i_s ranging from nanomolar to low affinity for the mMOR-1B4 isoform [45], [52]), which means that specific morphine concentrations are needed to activate a given receptor isoform. In addition, homo- and hetero-dimerisation of MOR1 subtypes (or DOR) induce switch in signaling [53].

Endogenous morphine may play a role in brain plasticity and development. At very low concentration (10^−9^ to 10^−14^ M), morphine increases neurite outgrowth in rat primary neuron cultures [54], as well as in the PC-12 tumor cell line [55], *via* a naloxone-independent pathway. In addition, MORs are involved in granule neuron genesis in the mouse cerebellum [56]. Recently, Zeng et al. demonstrated that morphine promotes the regeneration and synaptic reconstruction of the terminals of injured primary unmyelinated afferent fibers by an MOR-mediated, naloxone-dependent process [57]. Morphine has also been shown to modulate synaptic plasticity at hippocampal glutamatergic synapses [58]. Together, these data suggest that secreted endogenous morphine, at low concentrations, can act as a neurofactor involved in regulating the development of specific brain areas or in neuronal plasticity. Morphine activity at low concentrations was also observed in the present study, since the electrophysiological response of SH-SY5Y cells occurred at morphine and M6G concentrations as low as 10^−10^ M.

Previous reports have shown that PEBP is able to bind morphine [14] and M6G [15], suggesting that PEBP is an endogenous morphine/M6G binding protein. In the present study, PEBP immunoreactivity colocalized with that of morphine in basket cell nerve endings, which suggests the presence of PEBP-morphine complexes. Whether morphine is secreted in a complex with PEBP remains an open question.

### (*iv*) Presence of a specific catabolism process for morphine

Neurotransmitters are commonly inactivated in the synaptic gap by enzymes [59], or they are taken up by neurons and astrocytes for recycling or degradation [60]. Once inactivated inside the cell, the inactive product is released into the extracellular space. If morphine is a true neurotransmitter, it should be either inactivated or recycled.

Exogenously administered morphine is known to be catabolized in hepatocytes by enzymes of the UDP-glucuronosyl transferase 1A and 2B families (UGT) [61]. This leads to the formation of morphine-3-glucuronide (M3G; 90%) and M6G (10%). In contrast to the inactive M3G, M6G displays analgesic activity that is much more potent than morphine [15], [16]. In the rodent liver, the picture appears to be more complex because in rats, morphine is catabolized to both M6G and M3G [21], whereas only M3G is formed in mice [31].

In the brain, morphine uptake has been described in different cell types, including neurons [35]. Morphine is known to pass the blood brain barrier and to penetrate into the CNS more actively than M6G, probably because it is more hydrophilic [62]. Uptake mechanisms for morphine also appear to exist in non-neuronal cells, as shown by studies with radioactive morphine and human primary white blood cells [22], [35], [36], [37].

Interestingly, enzymes implicated in converting morphine into M3G (*i.e*., UGT1A enzymes) are expressed in primary neurons [19] and astrocytes [63]. Human and rat brain extracts, including those from the cerebellum, are able to synthesize labeled M3G and M6G from radiolabelled morphine [21], [22], proving that morphine catabolism/anabolism occurs in at least some brain structures. The uptake of released morphine may occur at the pre- or postsynaptic level in neurons as well as in astrocytes. Following uptake, morphine may be recycled in secretory organelles or converted into M3G by the UGT enzyme family present in neurons [64], [65] and astrocytes [63]. The resulting inactive M3G may then be transported from the cytoplasm into the extracellular space [31].

### Conclusion

Our present observations, together with previously published data, strengthen the proposal that endogenous morphine represents a new neurotransmitter or neuromodulator involved in signalling regulation. Further investigation is required to understand the precise role of endogenous morphine in brain physiology.

## Materials and Methods

### Animals

Experiments were performed on 37 day old laboratory-bred, adult male C57BL/6 mice weighing 30±3 g. Animals were given free access to food and water, with a 12 h light-dark cycle at a temperature of 22±2°C. All experiments were carried out in accordance with the European Community Council Directive (86/609/EEC) of November 24, 1986.

### Cell culture

The human SH-SY5Y cell line (ATCC number CRL-2266) was cultured for 5 days in 60 cm^2^ dishes in Dulbecco's Modified Eagle medium (Sigma Aldrich) supplemented with 10% (v/v) fetal bovine serum, 100 U/ml penicillin and 100 µg/ml streptomycin. The cells were incubated at 37°C in a wet atmosphere (relative humidity >95%) of 5% CO_2_ in air. The medium was changed every three days.

### Immunohistochemistry

#### Tissue preparation for immunocytochemistry studies

Mice were deeply anesthetized by intraperitoneal injection of 0.1 ml of a 5.6% (w/v) pentobarbital sodium solution (CEVA Santé Animale) and perfused transcardially with fixative solution using a peristaltic pump. For electron microscopy experiments, the fixative solution consisted of 4% formaldehyde (Sigma-Aldrich) in NaCl/Pi buffer (0.9% NaCl and 25 mM sodium phosphate, pH 7.4) with 0.25% glutaraldehyde (VWR). The same fixative solution was used for optical microscopy, except that glutaraldehyde was omitted. Fixative solutions were chilled, then injected for 10 min with a peristaltic pump at a flow rate of 10 ml/min. The brain was quickly removed and incubated for 2 hours at 4°C in the same fixative. Coronal and sagittal brain sections (80 µm thick) were cut with a vibratome (Leica VT 1000 S) and collected in Tris-buffered saline (TBS: 50 mM Tris-HCl, 0.9% NaCl, pH 7.4).

#### Cell preparation for immunocytochemistry studies

Cells cultured on glass coverslips were pre-fixed for 5 min with a solution of 4% (v:v) formaldehyde in NaCl/Pi buffer mixed with the culture medium at 25°C. Then, cells were fixed with 4% formaldehyde solution in NaCl/Pi buffer and permeabilized for 10 min with 0.1% (v/v) Triton X100 [15]. Glass coverslips were mounted on a glass slide with a drop of Mowiol 4-88.

#### Immunostaining

Immunostaining was performed on sections free-floating in TBS or on cells grown on coverslips as previously described [66]. Sections or slides were washed in TBS and incubated for 1 hour in bovine serum albumin diluted in TBS (3%, w/v) in order to saturate nonspecific immunoreactive sites. After six TBS washes of 5 min each, sections were incubated overnight in different antisera. Primary antibodies were used as follows: (*i*) mouse monoclonal antibodies (6D6, Aviva System Biology; dilution 1∶2000) or sheep polyclonal antibodies (AbD Serotec; dilution 1∶500) raised against morphine-like compounds (morphine, M3G, and M6G, based on supplier specifications and our own experiments, described below), (*ii*) goat polyclonal antibody raised against human and murine UGT2B [15], (*iii*) mouse monoclonal anti-glutamic acid decarboxylase (Chemicon; dilution 1∶1000), (*iv*) purified rabbit anti-PEBP antibody (dilution 1∶100) [13] and (*v*) mouse monoclonal antibody anti-CGA 5A8 (dilution 1∶500) [67].

After incubation with the primary antibody, sections or slides were washed six times with TBS (5 min) and specific secondary antisera were added for 2 h at room temperature, followed by six TBS washes (5 min). These secondary antisera were (*i*) HRP-conjugated donkey anti-mouse IgG (P.A.R.I.S.; dilution 1∶500), (*ii*) Alexa Fluor 488-conjugated donkey anti-sheep IgG (Molecular Probes; dilution 1∶2000), (*iii*) Alexa Fluor 568-conjugated donkey anti-goat (1∶2000), (*iv*) Cy5-conjugated donkey anti-sheep IgG (Jackson Immunoresearch Laboratories; dilution 1∶200) and (*v*) Cy3-conjugated donkey anti-mouse IgG (Jackson Immunoresearch Laboratories, dilution 1∶800).

Several controls were carried out to assess antibody specificity and nonspecific immunoreactivity. Primary antibodies were omitted, and each secondary antibody was tested individually or in a mixture in the presence of tissue sections or cells. Controls for morphine and UGT2B immunoreactivities were carried out by incubating the antibody with morphine (2 h, 25°C, 50∶1, w/w) or the corresponding blocking peptide (12 h, 4°C, 1∶5, w/w) [15], respectively, prior to immunocytochemistry experiments. Each antibody was also tested with the secondary antibody used for the second immunolabelling in order to determine whether interspecies cross-reactivity exists. Anti-morphine antibodies were also tested by ELISA in order to determine cross reactivity with morphine, M6G, and M3G, showing a specificity for morphine M6G and M3G. In order to assess whether morphine binds to proteins nonspecifically, extracts of SH-SY5Y and the mouse hippocampus were submitted to Western blot analysis; the results show that neither anti-morphine antibody labels proteins in these extracts. All experiments using anti-morphine antibodies were done in duplicate using mouse monoclonal and sheep polyclonal antibodies. The pattern of immunolabelling was similar each time.

### Light microscopy immunocytochemistry

Peroxidase activity was measured after a 20 min incubation in a freshly prepared solution of 4-chloro 1-naphtol (0.2 mg/ml) in TBS containing 0.006% (w/v) hydrogen peroxide. After washing with TBS, the sections were mounted in glycerol/TBS (1∶1, v/v) before analysis with a Leica DMRB microscope equipped with a digital camera (Axiocam, Zeiss).

### Electron microscopy immunocytochemistry

Peroxidase activity was detected with a freshly prepared solution of 0.025% (w/v) 3,3-diaminobenzidine tetrahydrochloride in TBS containing 0.006% (w/v) hydrogen peroxide. After washing with NaCl/Pi buffer, sections and slides were postfixed for 30 min with 2.5% glutaraldehyde in 0.1 M NaCl/Pi buffer (pH 7.4), and then with 1% (w/v) osmium tetroxide in 0.1 M NaCl/Pi buffer (pH 7.4) at 4°C for 1 hour. Sections were dehydrated in ethanol and embedded in Araldite resin [66]. Ultrathin sections were observed with a Hitachi H 7500 electron microscope without additional staining. Pictures were acquired with a Hamamatsu Digital camera (C 4742-95).

### Confocal microscopy observations

Immunofluorescent staining was analyzed with a Leica laser scanning microscope (TCS-SP2 invert) equipped with a plan apo 63× oil immersion lens. Tissue sections were subjected to optical serial sectioning to produce images in the X–Y plane. Each optical section was scanned eight times for brain sections and four times for cells to obtain an average image. Pictures were recorded digitally in a 512×512 pixel format. A look-up table (glowoverglowunder, Leica) ensured that the full dynamic range of the photomultipliers was used. Before each measurement, a series of sections was acquired through the vertical axis in order to choose the equatorial section.

### Anatomical and cellular distribution

Anatomical structures were identified under direct observation using the atlas and nomenclature of Paxinos and Watson [68]. Light microscopic sections were examined using a Leica DMRB brightfield microscope (objectives 1.6× to 20×).

### Isolation of exocytosed material from stimulated SH-SY5Y cells

SH-SY5Y cells were grown for 4 days in 24-well dishes (4×10^5^ cells per well) in DMEM with 10% fetal bovine serum (FBS, Sigma Aldrich). Prior to the experiments, they were washed twice (5 min each) with FBS-free DMEM at 37°C and incubated for 1 h in FBS-free DMEM at 37°C. Cells were then incubated with or without 100 µM nicotine in FBS-free DMEM for 24 h. The culture medium was collected and tested for morphine using a morphine-specific ELISA kit (Immunalysis Corporation; see below). Cadmium treatments (200 µM; Sigma Aldrich) were used to assess whether a Ca^2+^-dependent release mechanism exists.

Secretion efficiency was checked by Western blot analysis (see below) using a validated antibody raised against the conserved C-terminal part of chromogranin B (bovine CGB_614-626_ [69]), which serves as a secretion marker [27].

### Alkaloid and protein analysis

Prior to HPLC separation, Western blot analysis, or ELISA, scraped cells or homogenized brain areas were sonicated at 4°C (3×10 sec) in water containing protease inhibitor cocktail (Roche Diagnostics). The sonicates were centrifuged (30 min, 10000 *g*, 4°C), and the supernatant containing the intracellular material was used for protein analysis after protein quantification [15].

Endogenous alkaloids present in the tissue or cell homogenates were extracted as described in our previous study [15].

### Purification of alkaloids by reverse phase HPLC

Deproteinized or untreated samples corresponding to 375×10^6^ cells, as well as alkaloid standards, were purified using an Äkta purifier HPLC system (GE Healthcare Bioscience) as previously described [15]. The gradients of acetonitrile (ACN) are indicated on the chromatograms. Buffer A was 0.1% (v/v) in water, and buffer B was 70% acetonitrile and 0.09% , trifluoroacetic acid in water.

### Mass spectrometry

MS and MS-MS analyses were performed using electrospray mass spectrometry (ES-MS) on a Q-TOF II (Bio-Tech) in positive mode as previously described [15].

### Gel electrophoresis and Western blot analysis

Proteins were separated on SDS-PAGE gradient gels (4%–12% acrylamide; Criterion XT, BioRad) and electrotransferred onto polyvinyldifluorene membranes (GE Healthcare Bioscience) [13]. UGT2B enzymes were detected with a goat polyclonal antibody raised against the human UGT2B family (Santa Cruz Biotechnology, 65 kDa, dilution 1∶1,000), and signal was developed using HRP-conjugated anti-goat antisera (Santa Cruz Biotechnology, dilution 1∶50,000) [15]. Human hepatocyte extract (XenoTech) and human recombinant UGT2B7 (Sigma Aldrich) were used as positive controls. Chromogranin B (CGB) was detected using a rabbit polyclonal antibody raised against the conserved C-terminal part of chromogranin B (bovine CGB_614-626_ [69]), and the signal was developed using HRP-conjugated anti-rabbit antisera (Sigma Aldrich, dilution 1∶400,000)

### Morphine-specific ELISA

The morphine-specific ELISA kit from Immunalysis Corporation was used for the quantification of morphine present in culture medium (n = 6) and brain tissue extracts (n = 5). The specificity of the test for morphine was confirmed by testing different amounts of M6G, M3G, and codeine (0–25 ng/ml, data not shown). For all tests, morphine standards were diluted in the appropriate buffer.

### Statistical analysis of the secretion experiments

In order to assess the effect of nicotine stimulation on SH-SY5Y cells, morphine concentrations in the medium were subjected to *post hoc* analysis using a Mann-Whitney test. Our analysis included four different conditions: control, control+cadmium, nicotine, nicotine+cadmium. Each value was the result of six independent experiments (n = 6). Statistical data analysis was performed using MINITAB 13.20 (Minitab Inc.). Differences were considered to be statistically significant when the probability value was <0.01 (indicated by asterisks in the Figure).

### Total RNA isolation and Reverse-Transcription Polymerase Chain Reaction

Total RNA was extracted from SH-SY5Y cells using the NucleoSpin RNA II extraction kit (Macherey-Nagel). RNA purity was quantified by UV spectrophotometry at 260/280 nm.

Reverse-Transcription Polymerase Chain Reaction (RT-PCR) was performed with the One-Step RT-PCR kit (Qiagen) according to the manufacturer's protocol. Briefly, total RNA (100 ng) was retro-transcribed into cDNA for 30 min at 50°C; after inactivation of the transcriptase at 95°C for 15 min, PCR was performed for 35 cycles (1 min at 94°C, 1 min at the specific T_m_, 1 min at 72°C) followed by a final extension for 10 min at 72°C on a Mastercycler personal (Eppendorf).

Forward and reverse primers specific to human MOR1 (5′-3′ ACCAACATCTACATTTTCAACCTT and 5′-3′ CAGTACCAGGTTGGATGAGAG) were designed to amplify a 376-bp fragment of the region common to all MOR1 splice variants (OligoExplorer 1.2, Gene Link).

Human UGT2B7 forward and reverse primers (5′-3′ TCCACGAGCATCTTCGAGA and 5′-3′ ATACTGGAAGCACATGCCC) were designed to amplify a 462-bp fragment specific to UGT2B7, (OligoExplorer 1.2, Gene Link).

Amplification of GAPDH RNA was used as a positive control in RT-PCR reactions. PCR products (one-fifth of the PCR reaction) were loaded on an ethidium bromide-stained agarose gel (0.8%) and visualized by UV illumination using the GeneSnap system (Syngene).

Reactions were carried out using synthesized oligonucleotides (6 µM final concentration) at a T_m_ of 51°C for MOR1 and UGT2B7.

### Patch-clamp experiments on SH-SY5Y cells

For patch-clamp recording, SH-SY5Y cells were maintained in culture (DMEM+10% FCS and 50 U/ml penicillin, 50 µg/ml streptomycin) at low density (about 2×10^4^ cells/ml) in 35 mm dishes and subjected to recording experiments 3–8 days after plating. M6G-induced responses were measured either in the cell-attached or whole-cell configuration of the patch-clamp technique [70] using an Axopatch-B200 amplifier (Axon Instruments). For whole-cell recording, the recording pipette had a 2–4 MΩ resistance when filled with recording solution (131 mM K-gluconate, 10 mM HEPES, 5.5 mM EGTA, 1 mM CaCl_2_, 2 mM MgCl_2_, 3 mM NaCl 5 mM KCl, 0.1 mM ATP, and 0.1 mM GTP). Isolated SH-SY5Y cells were selected and recorded under continuous perfusion with control medium (132 mM NaCl, 2 mM KCl, 1 mM CaCl_2_, 2 mM MgCl_2_, 1 mM Na-Gluconate, 10 mM HEPES and 10 mM d-glucose). The pH of the pipette and bath medium was adjusted to 7.2 with KOH and 7.4 with NaOH, respectively. For cell-attached recordings, the pipette was filled with bath medium. Voltage command and current trace digitization were achieved using the Digidata 1322A card interface (Axon Instruments) and Pclamp software (Axon instrument). Current traces were low-pass filtered at 2 kHz before digitization at 5 kHz.

Drugs were diluted to the desired concentrations in the bath medium and applied to the recorded cell through a multi-barrel perfusion system (RCS-160 rapid solution exchanger, Bio-Logic). Each barrel had a 1 mm inner diameter, and the selected perfusion tube was placed about 50 µm from the recorded cell. Drug application began when the selected tube was positioned in front of the cell. Naloxone antagonist was purchased from Sigma Aldrich.
